# Pool Boiling Heat Transfer Characteristics of Hydrophobically Modified TiO_2_@Carbon Nanotube Composite Nanofluids

**DOI:** 10.3390/nano16030152

**Published:** 2026-01-23

**Authors:** Yongli Wu, Zhongmin Lang, Gangqiang Wu, Ying Yu, Panpan Yan, Yufei Yang, Zeyu Zhang

**Affiliations:** 1 College of Chemical Engineering, Ordos Institute of Technology, Ordos 017000, China; wuyongli2025@oit.edu.cn (Y.W.);; 2 Inner Mongolia Duanli New Energy Research Institute Co., Ltd., Baotou 014010, China

**Keywords:** TiO_2_, hydrophobic modification, carbon nanotube, nanofluids, enhanced heat transfer

## Abstract

To tackle challenges including excessive initial boiling superheat and low heat transfer coefficients inherent in conventional working fluids, hydrophobic-modified TiO_2_@carbon nanotube (MWCNT) composite nanofluids were fabricated. Subsequently, the boiling heat transfer mechanisms were systematically investigated and visually verified. Hydrophobic TiO_2_ nanofluids exhibit enhanced stability, whereas hydrophobic TiO_2_@MWCNTs composite nanofluids demonstrate improved thermal conductivity. At a mass ratio of hydrophobic-modified TiO_2_ to MWCNTs of 2:1, the optimal heat transfer performance was attained, with a 31.6% increase in heat transfer coefficient (HTC) and a 46.5% increase in critical heat flux (CHF) density relative to hydrophobic-modified TiO_2_ nanofluids. Composite nanofluids exert effective regulation over bubble kinetic parameters: hydrophobic nanoparticles increase vaporization core density, reduce bubble nucleation energy barriers, and mitigate initial boiling superheat. Benefiting from the superior thermal conductivity and mechanical properties, MWCNTs remarkably promote heat transfer efficiency. The synergistic effect between the two components enables the concurrent enhancement of HTC and CHF, thus highlighting the promising application potential of hydrophobic-modified TiO_2_@MWCNTs composite nanofluids in intensifying pool boiling heat transfer.

## 1. Introduction

In recent years, energy issues have become increasingly prominent, and enhancing energy efficiency has emerged as the primary countermeasure to address the energy crisis [1]. As power densities of electronic components continue to rise, the thermal management performance of electronic devices requires further optimization, making thermal management an urgent and critical challenge that demands immediate resolution. Boiling heat transfer, as a notably efficient heat transfer method, achieves remarkable heat dissipation rates at low superheat, thereby rendering it widely utilized in industrial refrigeration, power generation, and electronic chip cooling [2,3].

Currently, conventional heat transfer fluids (e.g., water, alcohols, and mineral oils) possess inherent limitations in practical engineering applications due to the relatively low heat transfer coefficients [4,5,6,7]. Tailoring the composition of working fluids to enhance pool boiling heat transfer represents a viable and effective strategy for tackling the challenges of high heat flux dissipation [6,8,9,10]. Through an investigation into the enhancement effects of diverse working fluids on pool boiling heat transfer, we persistently probe the boundaries of heat transfer performance to attain higher critical heat flux (CHF) and heat transfer coefficient (HTC) [11,12]. Nanofluids, utilized as working fluids in boiling heat transfer, possess enhanced thermal conductivity and superior convective heat transfer performance, thereby demonstrating outstanding heat transfer augmentation capabilities [13,14,15,16,17,18].

Jamialahmad [19] investigated the saturated pool boiling heat transfer performance of water-based CuO nanoparticles (40 nm) nanofluids. The findings revealed that the CHF attained a maximum value at a concentration of 100 mg/L, corresponding to a 92% enhancement relative to deionized water. Shaafi [20] examined the pool boiling heat transfer performance of three distinct nanofluids (SiO_2_, Al_2_O_3_, and ZrO_2_) with volume concentrations spanning 0.001 to 0.1 vol%. The findings revealed that as the nanofluid concentration increased, the slope of the boiling curve steepened progressively. Specifically, at the maximum concentration of 0.1 vol%, the heat transfer coefficient (HTC) of the Al_2_O_3_-based nanofluid surpassed that of deionized water by 116.6%, the SiO_2_-based nanofluid exceeded that of deionized water by 84%, and the ZrO_2_-based nanofluid achieved a 2% enhancement relative to that of deionized water. Phan [21] investigated the saturated pool boiling heat transfer performance on hydrophobic-modified surfaces (solid–liquid contact angle: 20–110°). The findings revealed that during the incipient boiling stage, hydrophobic-modified surfaces facilitated rapid bubble detachment and departure, thereby enhancing the overall boiling heat transfer efficiency. Collectively, the results demonstrate that hydrophobic-modified surfaces exert a substantial influence on saturated pool boiling heat transfer performance. Analytical findings reveal that nanofluids can reduce the bubble nucleation energy barrier and enhance the overall heat transfer efficiency of pool boiling systems. During the incipient bubble growth stage, the thermal resistance associated with bubble nucleation and formation is diminished, thereby facilitating rapid bubble detachment and departure and consequently yielding a significant improvement in saturated pool boiling heat transfer performance. Current formulations of single-component nanofluid exhibit several critical limitations, including suboptimal heat transfer efficiency, non-uniform nanoparticle dispersion [22,23,24,25,26], and pronounced particle agglomeration upon long-term deployment [27,28,29,30]. Therefore, the selection of materials possessing superior thermal conductivity and favorable dispersibility has emerged as a key research priority in the field of nanofluid-mediated heat transfer.

To improve and optimize the thermophysical properties and saturated pool boiling heat transfer performance of nanofluids, researchers have proposed the fabrication of composite nanofluids via the homogeneous mixing and dispersion of two or more distinct nanoparticle types in a base fluid [31]. Wei [32] investigated the pool boiling heat transfer performance of oil-based composite SiC-TiO_2_ nanofluids. The results demonstrate that the heat transfer coefficient of pure heat transfer oil decreases with rising temperature. In contrast, the composite nanofluid markedly enhances boiling heat transfer performance and exhibits superior heat transfer characteristics compared to single-component nanofluids (SiC/heat transfer oil or TiO_2_/heat transfer oil). Nabil [33] developed a correlation model to predict the thermal conductivity of fluids containing TiO_2_-SiO_2_ nanoparticles, in which the composite thermal conductivity exhibited a maximum enhancement of 22.8%. Analytical findings indicate that composite nanofluids can provide abundant nucleation sites for onset of nucleate boiling (ONB), enhance liquid reflux capacity, improve surface roughness on heating surfaces, restrict bubble growth radius, and enhance CHF.

TiO_2_ nanofluids are extensively utilized in the energy sector due to the excellent thermal conductivity and favorable safety profile. However, the high surface energy renders the fluid stability prone to disruption, which can lead to nanoparticle agglomeration [34,35,36]; hydrophobic coating technology exhibits notable advantages in specialized applications, such as cooling systems and adsorption processes, yet it entails a relatively high cost and may lead to abnormal fluid viscosity, thereby compromising heat transfer efficiency [15]. In thermal applications, MWCNT nanofluids outperform conventional nanofluids in heat transfer performance—an advantage stemming not from exceptionally high thermal conductivity but from their unique tubular nanostructure. However, strong van der Waals interactions between MWCNTs result in easy entanglement and agglomeration, rendering it challenging to sustain a uniformly dispersed state over prolonged periods, even with dispersion treatments [37,38,39]. In this study, hydrophobic TiO_2_ nanoparticle-decorated MWCNT composite nanofluids were developed. TiO_2_ nanoparticles exhibit excellent thermal and chemical stability, and the hydrophobicity can be tailored via surface modification. MWCNTs act as ideal thermal interface materials owing to their ultra-high thermal conductivity, large specific surface area, and excellent mechanical properties. The hydrophobic TiO_2_@MWCNT composite exerts a synergistic enhancement effect on boiling heat transfer performance: MWCNTs impart ultra-high thermal conductivity, while hydrophobic TiO_2_ modulates the heating surface’s wettability. TiO_2_ nanoparticles and MWCNTs achieve uniform dispersion, which significantly mitigates particle agglomeration and modulates the nucleation, growth, and detachment behavior of bubbles during boiling. This uniform dispersion enables precise modulation of deposition phenomena on heating surfaces, thereby laying a structural basis for heat transfer enhancement. This multi-mechanism synergistic heat transfer enhancement strategy provides novel insights into the development of high-performance cooling fluids and holds considerable potential for practical applications.

## 2. Materials and Methods

### 2.1. Materials

TiO_2_ nanoparticles (15 nm, purity > 99%, purchased from Hangzhou Zhitai Purification Technology Co., Ltd., Hangzhou, China); MWCNTs (8–15 nm, purity > 95%, purchased from Nanjing Xianfeng Nanomaterials Technology Co., Ltd., Nanjing, China); perfluorodecyltrimethoxysilane; ethanol (≥99.8%); deionized water (self-prepared).

### 2.2. Preparation Composite Nanofluids

**Figure 1:**
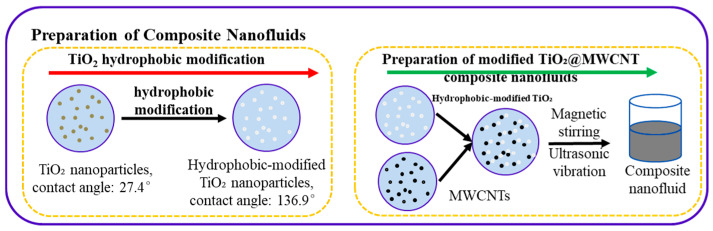
Hydrophobic TiO_2_@MWCNT nanofluid preparation process.

**Figure 2:**
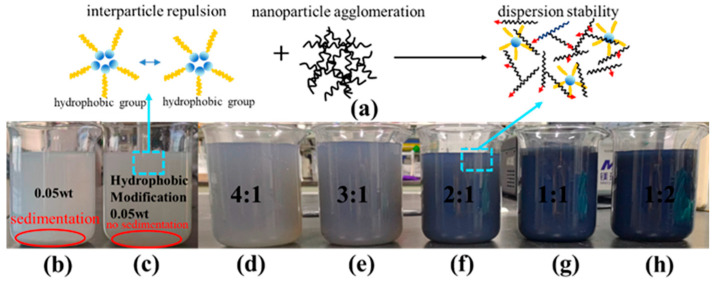
TiO_2_ nanofluids before and after modification and stability testing of composite nanofluids: (**a**) mechanism diagram of composite nanofluid; (**b**) TiO_2_ nanofluid; (**c**) hydrophobically modified TiO_2_ nanofluid; (**d**–**h**) 4:1–1:2 composite nanofluid.

In this study, perfluorodecyltrimethoxysilane, ethanol, and deionized water were mixed at a volume ratio of 1:49:50, followed by stirring at room temperature for 6 h to ensure complete hydrolysis. Subsequently, TiO_2_ nanoparticles were thoroughly immersed in the hydrolyze solution and reacted for 5 h. After the reaction, the mixture was transferred to an oven and dried at 120 °C for 90 min, yielding modified TiO_2_ nanoparticles, which were then mixed with MWCNTs and deionized water. The mixture was stirred with a magnetic stirrer (Model SH-2) for 5 h, and then sonicated in an ultrasonic cleaner (Shenzhen Yujie Cleaning Equipment Co., Ltd. Model AK-404S, Shenzhen, China) for 30 min, affording a composite nanofluid with excellent stability. The process flow is depicted in Figure 1. Static contact angle measurements for TiO_2_ nanoparticles pre- and post-modification yielded values of 27.4° and 136.9°, respectively, confirming the successful hydrophobic modification of the TiO_2_ nanoparticles. Transmission electron microscopy (TEM) images of the composite nanofluid confirm that both TiO_2_ and MWCNTs retain intact structures and exhibit uniform distribution in the fluid. It can be verified from the experimental results in Figure 2 and Figure 5 that the prepared composite nanofluid exhibits good stability (no deposition occurred, and the hydrophobicity remained unchanged before and after boiling). To verify the accuracy and repeatability of the experiment, the smooth copper surface was rinsed three times with deionized water and anhydrous ethanol both before and after the experiment to ensure that the surface was free of impurities.

Figure 2 presents the underlying heat transfer mechanism of the modified TiO_2_@MWCNT composite nanofluid and the image of the nanofluid following 20 days of static storage. As the MWCNT content increased, the color of the nanofluid solution deepened owing to the intrinsic dark carbonaceous nature of MWCNTs, with no adverse effect on its dispersion stability. Hydrophobic nanoparticles modulate surface energy, inducing interparticle repulsion and thereby significantly enhancing nanofluid dispersion stability. MWCNTs possess a large specific surface area and ultra-high thermal conductivity but tend to agglomerate in fluid media due to strong van der Waals interactions between nanotubes. Hydrophobic nanoparticles augment interparticle repulsion via surface modification, thereby inhibiting the formation and growth of nanoparticle agglomeration. MWCNTs enhance the heat transfer performance of hydrophobic nanofluids and optimize the bubble growth process during boiling. The synergistic integration of these two components tailors the internal microstructure of the nanofluid, improving its dispersion stability and thereby sustaining high heat transfer efficiency. Following 20 days of static storage, TiO_2_ nanoparticles and MWCNTs within the composite nanofluid maintained uniform dispersion, with no sedimentation detected at the bottom of the storage beaker. Analysis reveals that hydrophobic modification generates an organic adsorption layer on the surface of TiO_2_ nanoparticles, effectively mitigating nanoparticle agglomeration and thus verifying the composite nanofluid’s superior dispersibility and long-term stability.

### 2.3. Experimental Setup

**Figure 3:**
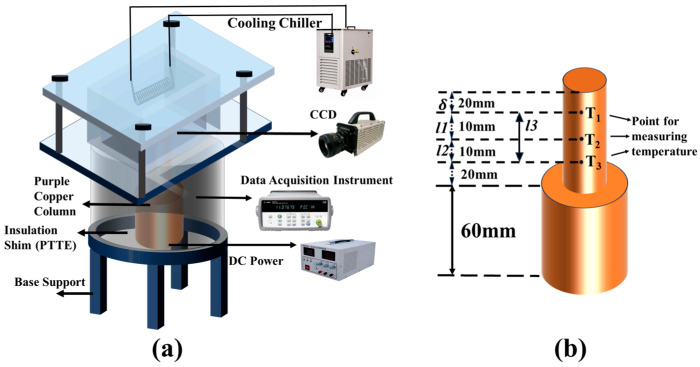
Schematic of pool boiling setup: (**a**) Stereogram of the test platform; (**b**) Plan diagram of the test chamber.

As shown in Figure 3, the experimental apparatus comprises a square quartz vessel, a heating system, a condensation reflux system, a temperature measurement system, and a data acquisition system. An electric heater was inserted at the base of the thermally conductive copper column to supply stable heat flux; high-precision thermocouples were embedded sequentially along the lateral surface of the column for real-time temperature monitoring during the experiment, while an additional thermocouple was immersed in the working fluid to measure its bulk temperature. A CCD high-speed camera and recording system (Photron FASTCAM/SA/X2 Viewer, Photron, Tokyo, Japan) were utilized to observe and record bubble dynamics.

### 2.4. Computing Method

The Fourier steady-state differential equation for the heat transfer coefficient was used to determine the heat flux density and surface temperature of the heat transfer surface. All temperature measurements were recorded using type T thermocouples. The heat flux density (q) of the heat-conducting column was calculated using Equation (1):

(1) $$q=\frac{U\times I\times 1000}{A}$$

where q is the heat flux, kW/m^2^; U is the voltage, V; I is the current, A; and A is the heat transfer area, m^2^.

The thermocouple-measured temperature can be used to compute the $T_{W}$:

(2) $$T_{W}=T_{ave}-q\left(\frac{l_{s}}{k_{s}}+\frac{l_{c}}{k_{c}}\right)$$

where $T_{ave}$ is the heat-conductivity column’s surface temperature, $l_{s}$ and $l_{c}$ are the thicknesses of the solder paste and test sample, respectively, and $k_{s}$ and $k_{c}$ represent the coefficients of heat transfer coefficient of the solder paste and test sample, respectively. $T_{ave}$ is calculated by Equation (3):

(3) $$T_{ave}=T_{1}-\frac{\delta }{3}\left(\frac{T_{2}-T_{1}}{l1}+\frac{T_{3}-T_{2}}{l2}+\frac{T_{3}-T_{1}}{l3}\right)$$

where *δ* signifies the distance between the uppermost measurement point of the heat-conducting copper column and its top, l1, l2, and l3 denote the distances between thermocouples $T_{1}$ and $T_{2}$, $T_{2}$ and $T_{3}$, and $T_{1}$ and $T_{3}$, respectively.

The ΔT is determined by the temperature difference between the heat transfer surface and the working fluid, given as

(4) $$\Delta T=T_{W}-T_{sat}$$

where $T_{sat}$ is the working fluid temperature, and $T_{W}$ is the heat transfer surface temperature.

Meanwhile, the boiling h is

(5) $$h=\frac{q}{∆T}$$

### 2.5. Error Analysis

The measurement uncertainties of voltage and current for the experimental test rig were ±0.1 V and ±0.1 A, respectively; the thermocouple had a temperature measurement uncertainty of ±0.2 K. The maximum experimental uncertainty associated with heat loss was 2.4%, that of the contact angle measuring instrument was 2%, the thermal conductivity measurement uncertainty was 4.1%, and the uncertainty of the effective heat transfer area was 0.012%.

(6) $$E_{r}=\frac{\Delta \lambda }{\bar{\lambda }}\times 100\%$$

The calculation method for heat flux error was as follows:

(7) $$\frac{∆q}{q}=\sqrt{{\left(\frac{∆T}{T}\right)}^{2}+{\left(\frac{∆k}{k}\right)}^{2}+{\left(\frac{∆Q}{Q}\right)}^{2}+{\left(\frac{∆A}{A}\right)}^{2}}\leq \frac{∆T}{T}+\frac{∆k}{k}+\frac{∆Q}{Q}+\frac{∆A}{A}$$

=0.15%+1.1%+2.4%+0.012%=3.66%

Equation (8) yielded the wall surface superheat error as follows:

(8) $$\frac{∆\left(T_{0}-T_{l}\right)}{T_{0}-T_{l}}=\sqrt{{\left(\frac{∆q}{q}\right)}^{2}+{\left(\frac{∆k}{k}\right)}^{2}}\leq \frac{∆q}{q}+\frac{∆k}{k}=3.66\%+1.1\%=4.76\%$$

Equation (9) yielded the relative error of the heat transfer coefficient as follows:

(9) $$\frac{\Delta h}{h}=\sqrt{{\left(\frac{\Delta q}{q}\right)}^{2}+{\left(\frac{\Delta T_{i}}{T_{i}-T_{w}}\right)}^{2}}\leq \frac{\Delta q}{q}+\frac{\Delta T_{i}}{T_{i}-T_{w}}$$

=3.66%+1.2%=4.86%

Measured uncertainties for heat flux density, wall superheat, and boiling heat transfer coefficient were 3.66%, 4.76%, and 4.86%, respectively.

For thermal performance under high heat flux density (i.e., CHF enhancement and delayed film boiling transition): the CHF improvement rate was 40 times the 3.66% relative uncertainty. For HTC enhancement over the entire heat flux range: the HTC improvement rate was 44 times the 4.86% relative uncertainty. Even accounting for the maximum potential measurement error, its CHF remained far higher than that of deionized (DI) water, verifying that delayed film boiling transition (boiling crisis mitigation) at high heat flux density is an inherent property of the composite nanofluid, with the resulting performance gain unaffected by measurement uncertainty.

In conclusion, all key findings of the present study, including the improved boiling heat transfer performance of the modified TiO_2_@MWCNT composite nanofluid and the associated bubble dynamics regulation mechanism, are experimentally credible, where the observed effects far exceed the uncertainties involved in the experimental measurements.

## 3. Results and Discussion

### 3.1. Composite Nanofluidic Structures and Characterization

**Figure 4:**
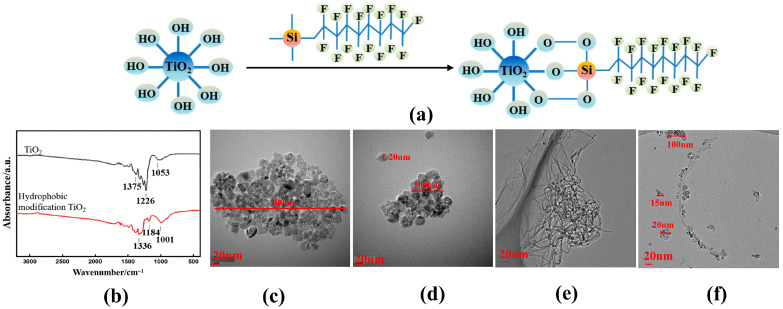
Infrared spectra and TEM images of TiO_2_ particles before and after modification: (**a**) schematic diagram of hydrophobic modification of TiO_2_; (**b**) infrared spectra of TiO_2_ nanofluids before and after modification; (**c**,**d**) TEM images of TiO_2_ nanofluid before and after modification; (**e**) TEM images of MWCNT nanofluid; (**f**) TEM images of TiO_2_@MWCNT nanofluid.

Figure 4a illustrates the schematic of the modification mechanism for hydrophobic TiO_2_ nanoparticles. The methoxy group (–OCH_3_)—a readily hydrolyzable moiety on the silane reagent—undergoes hydrolysis to yield reactive silanol groups (–Si-OH), which then participate in a sequential "hydrolysis–condensation" reaction to form covalent bonds with hydroxyl groups on the TiO_2_ nanoparticle surface. This process displaces the high-surface-energy polar hydroxyl groups on the nanoparticle surface with low-surface-energy non-polar perfluorodecyl groups, transforming the nanoparticle surface from hydrophilic–polar to hydrophobic–nonpolar and thereby accomplishing hydrophobic modification. Figure 4b shows the FTIR spectra of the TiO_2_ nanofluid before and after modification. Comparison of the spectra reveals that the characteristic –OH stretching vibration absorption peaks at 1226 cm^−1^ and 1184 cm^−1^ are markedly reduced in intensity. This phenomenon arises from the replacement of hydroxyl (–OH) groups on the TiO_2_ nanoparticles by hydrophobic functional groups via covalent bonding stabilization [40], further validating the hydrophobic modification mechanism. Analysis of TEM images revealing morphological changes in TiO_2_ nanofluids before and after modification shows that the unmodified TiO_2_ nanofluid (Figure 4c) exhibited blurred particle boundaries and dense agglomeration. Isolated particles were visible only in a few regions, with an individual particle size of 10–15 nm; however, agglomerates formed via particle aggregation reached sizes of 300–500 nm. In contrast, the hydrophobic TiO_2_ nanofluid (Figure 4d) exhibited relatively uniform particle sizes, distinct particle boundaries, and increased interparticle spacing. Numerous individually dispersed nanoparticles (size: 20–100 nm) were observed, and the overall agglomerate size was smaller than that of the hydrophilic TiO_2_ nanofluid. These observations confirm that hydrophobic modification effectively mitigated TiO_2_ nanoparticle agglomeration. Figure 4e presents the transmission electron microscopy (TEM) image of the MWCNT nanofluid. The MWCNTs exhibited pronounced entanglement and interweaving, forming dense agglomerates, with a small fraction of individual nanotubes protruding from cluster peripheries. This observation indicates that the MWCNT nanofluid possesses moderate dispersion stability that warrants further optimization to mitigate inter-tube aggregation. Figure 4f shows the transmission electron microscopy (TEM) image of the TiO_2_@MWCNTs nanofluid. The one-dimensional tubular structures observed were multi-walled carbon nanotubes (MWCNTs), with an outer diameter of ~10–20 nm and lengths extending up to several hundred nanometers. The MWCNTs exhibited favorable overall dispersion, with no large-scale entangled clusters and only localized mild interweaving, indicating that hydrophobic TiO_2_ nanoparticles effectively suppress MWCNT entanglement in this system. The spherical, bright white particles (15–20 nm in diameter) are TiO_2_ nanoparticles, which uniformly adhered to the MWCNT sidewalls and partially filled the inter-tube gaps. No large, free-standing TiO_2_ aggregates were detected, demonstrating an excellent loading and binding state.

**Figure 5:**
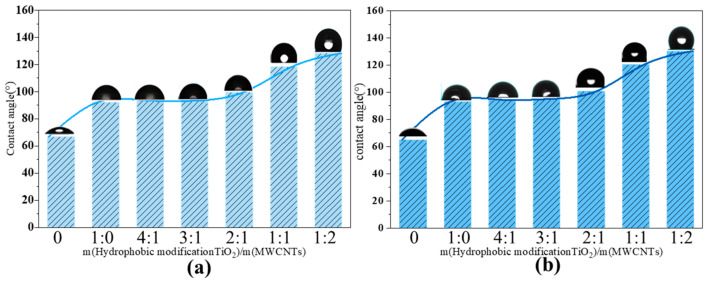
Contact angle of modified TiO_2_@MWCNT composite nanofluid: (**a**) contact angle before pool boiling; (**b**) contact angle after pool boiling.

The test was conducted using a contact angle measuring instrument produced by the German company Dataphysics (model: OCA 20; Dataphysics, Filderstadt, Germany). Contact angle testing can evaluate the hydrophobic modification effect of nanofluids. Triplicate measurements were conducted for each sample, and the corresponding average values are presented in the figure. When TiO_2_@MWCNT composite nanofluids with different proportions but a total concentration of 0.05% were dropped onto a smooth copper substrate, the resulting changes in the contact angle are shown in Figure 5. Before and after boiling, the contact angle remained essentially constant, indicating that there were no instability issues, such as aggregation or detachment of the nanophase, in this system. As the MWCNT ratio increased, the static contact angle of the composite nanofluid correspondingly increased. The modified TiO_2_@MWCNT composite nanofluid (4:1) exhibited a contact angle of 128.3°, representing an improvement of 36.1° over the modified TiO_2_ nanofluid. Based on the Cassie–Baxter model [41], the synergistic interplay between surface roughness and low surface energy can further enhance hydrophobicity. Modified TiO_2_ nanoparticles serve as a low-surface-energy hydrophobic matrix, whereas the porous network structure of MWCNTs increases surface roughness. This synergistic combination augments the hydrophobicity of the nanofluid and concurrently lowers the Gibbs free energy required for bubble nucleation, thereby reducing the boiling point of the hydrophobic nanofluid.

**Figure 6:**
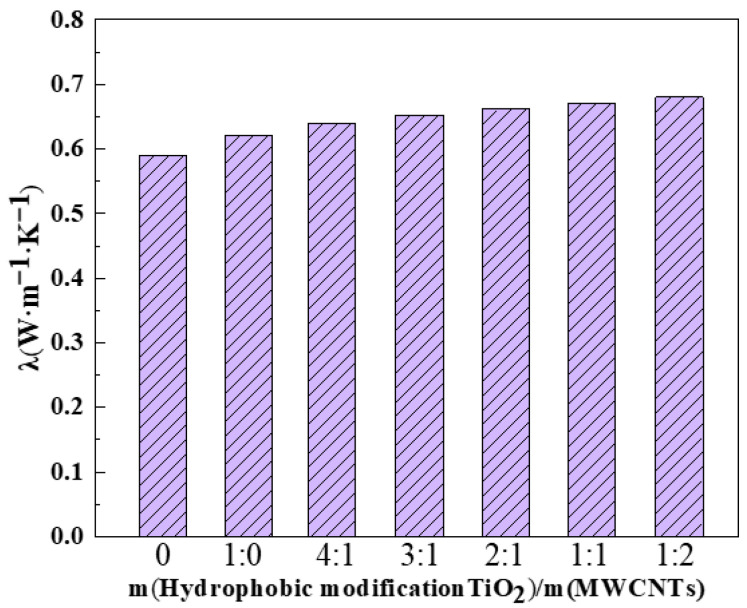
Thermal conductivity of modified TiO_2_@MWCNT composite nanofluids.

Thermal conductivity is a key parameter governing the heat transfer performance of nanofluids, and the corresponding measurement results are presented in Figure 6. The thermal conductivity of the as-prepared TiO_2_ nanofluids was determined via the transient hot wire method using a TC3000L thermal conductivity meter (Xi’an Xiaxi Electronic Technology Co., Ltd., Xi’an, China). Triplicate measurements were conducted for each sample, and the corresponding average values are presented in the figure. The modified TiO_2_@MWCNT composite nanofluid exhibited higher thermal conductivity than the single-component modified TiO_2_ nanofluid. Within a certain mass fraction range of MWCNTs, the boiling heat transfer coefficient increased with increasing MWCNT content. The modified TiO_2_ nanofluid achieved a thermal conductivity of 0.623 W·m^−1^·K^−1^, representing a 5.6% enhancement relative to the reference (deionized water). Among all samples, the modified TiO_2_@MWCNT composite nanofluid with a mass ratio of 1:2 (TiO_2_: MWCNTs) exhibited the maximum thermal conductivity of 0.681 W·m^−1^·K^−1^, corresponding to a 5.8% enhancement compared to the single-component modified TiO_2_ nanofluid. Analysis reveals that at higher MWCNTs concentrations, the ultra-high thermal conductivity of MWCNTs enhanced the thermal conductivity of the TiO_2_@MWCNT composite nanofluids. However, as the MWCNT concentration continued to increase, nanoparticle agglomeration became prevalent, attenuating the heat transfer enhancement efficacy of the nanofluid. Consequently, selecting TiO_2_@MWCNT composite nanofluids with an optimal mass ratio is critical to optimizing boiling heat transfer performance.

**Figure 7:**
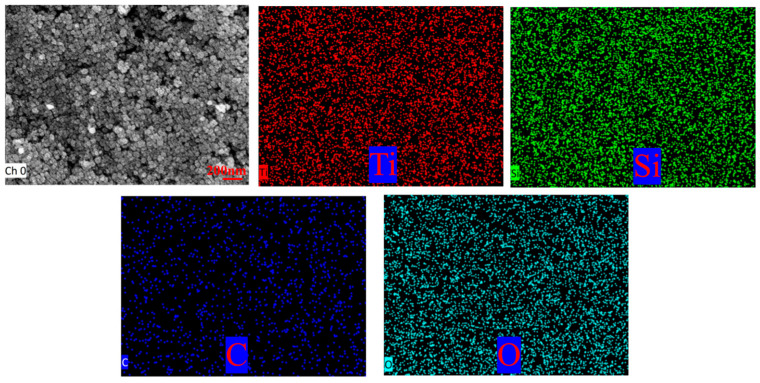
TEM images and energy spectrum tests of composite nanofluids.

Figure 7 presents transmission electron microscopy (TEM) images and energy-dispersive spectroscopy (EDS) analysis of the modified TiO_2_@MWCNTs composite nanofluid. No prominent agglomerates were observed, where hydrophobic TiO_2_ nanoparticles and MWCNT tubular structures display uniform dispersion throughout the fluid matrix. Energy-dispersive X-ray spectroscopy (EDS) characterization reveals that Ti and O elements derive from titanium dioxide (TiO_2_), Si elements originate from hydrophobic functional groups, and C elements stem from MWCNTs. The Si element was homogeneously distributed across the entire observation area without local enrichment or absence, indicating that the hydrophobic modifier was uniformly grafted onto the surface of TiO_2_ nanoparticles, with no partially unmodified nanoparticles observed. This ensured the uniformity of surface wettability for TiO_2_ nanoparticles, and the TiO_2_ nanoparticles modified with hydrophobic functional groups achieved homogeneous dispersion in the MWCNT composite system and the fluid matrix, which further corroborates the results of non-agglomerated and homogeneous dispersion obtained from TEM morphological characterization.

### 3.2. Pool Boiling Heat Transfer Performance Testing and Analysis

**Figure 8:**
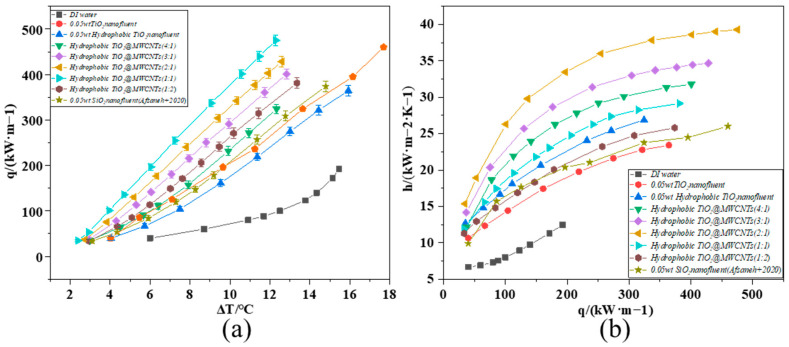
Composite nanofluid boiling heat transfer performance curve ((**a**): q varies with ∆T, (**b**): h varies with q) [42].

To investigate the influence of composite nanofluid composition on heat transfer performance, modified TiO_2_@MWCNT composite nanofluids with varying ratios were prepared and subjected to pool boiling heat transfer tests under atmospheric pressure. Figure 8a shows the CHF versus wall superheat curve. To clarify the effect of composite nanofluid components on heat transfer performance, modified TiO_2_@MWCNT composite nanofluids with different mass ratios and a total concentration of 0.05 wt% were prepared, and pool boiling heat transfer experiments were conducted on them under atmospheric pressure. Equations (10) and (11) reveal that the bubble nucleation energy barrier exhibited an inverse correlation with the contact angle: the larger the contact angle, the lower the bubble nucleation energy barrier. The TiO_2_@MWCNT composite nanofluid exhibited a contact angle increase of 10° relative to the modified TiO_2_ nanofluid, while its bubble nucleation energy barrier decreased by 37.91%. This offers theoretical corroboration for the aforementioned experimental results.

(10) $$f\left(\begin{array}{c} \theta \end{array}\right)=\frac{2+3cos\theta -{cos}^{3}\theta }{4}$$

(11) $$\Delta E_{f}=\left(\begin{array}{c} R^{*},\theta \end{array}\right)=\frac{4\pi R^{*2}}{3}\sigma \times f\left(\begin{array}{c} \theta \end{array}\right)$$

where $\begin{array}{c} \theta \end{array}$ is the contact angle, °; $R^{*}$ is the radius of bubble detachment, m; σ is surface tension, N/m.

At the same wall superheat, hydrophobic nanoparticles lower the energy barrier for bubble nucleation, thus effectively reducing the ONB and increasing the nucleation density; MWCNTs with UHTC further refine heat transfer pathways. The composite nanofluid formulation mitigated abrupt interparticle collisions during boiling, which inhibited bubble coalescence and thus improved the CHF. The modified TiO_2_@MWCNT composite nanofluid achieved a maximum CHF of 475.1 kW/m^2^ at a TiO_2_/MWCNTs mass ratio of 2:1. Analysis reveals that dispersed TiO_2_@MWCNT nanoparticles augmented microscale turbulence in the working fluid, significantly reducing interfacial thermal resistance. Furthermore, hydrophobic modification effectively alleviated nanoparticle agglomeration, delaying the nucleate-to-film boiling transition and thus boosting the CHF markedly. When the TiO_2_: MWCNTs mass ratio was below 1:2 (i.e., MWCNT content was twice that of TiO_2_), the flexible tubular structure of MWCNTs induced nanoparticle agglomeration, weakened interparticle Brownian motion, and impaired the continuity and stability of the heat transfer interface, leading to a significant deterioration in heat transfer performance. Therefore, optimizing the formulation ratio maximizes the synergistic effect between the nucleation-modulating capability of hydrophobically modified TiO_2_ nanoparticles and the ultra-high thermal conductivity of MWCNTs.

Figure 8b illustrates the evolution of the boiling HTC as a function of heat flux density. Experimental findings demonstrate that the composite nanofluid pool boiling system exhibited a markedly enhanced HTC compared to DI water. Pronounced discrepancies in HTC were observed across the tested working fluids: DI water reached a maximum HTC of 12.46 kW·m^−2^·K^−1^, whereas the hydrophobic-modified TiO_2_ nanofluid (0.05 wt%) achieved a peak HTC of 26.87 kW·m^−2^·K^−1^. The HTC of the modified TiO_2_@MWCNT composite nanofluid increased with an increasing proportion of MWCNTs relative to modified TiO_2_, reaching an optimum HTC of 39.28 kW·m^−2^·K^−1^ at a mass ratio of modified TiO_2_ to MWCNTs of 2:1. Analytical results demonstrate that modified TiO_2_ nanoparticles effectively alleviated particle agglomeration, ensuring uniform dispersion in the base fluid. Meanwhile, the superior thermal conductivity of MWCNTs facilitated the formation of efficient heat transfer pathways. The synergistic effect of these two components substantially reduces thermal resistance, optimizes heat transfer conditions at the heating surface-fluid interface, and further enhances boiling heat transfer performance.

The performance advantage of the composite nanofluid was more evident in the low superheat region (ΔT: 10 °C): the SiO_2_ nanofluid yielded a heat flux of 280–300 kW·m^−2^, while the TiO_2_@MWCNT composite system achieved 320–350 kW·m^−2^, corresponding to a 14–21% enhancement [42]. This result confirms that the composite nanofluid enabled higher heat flux at a reduced temperature difference, reflecting more efficient boiling initiation. Notably, the SiO_2_ nanofluid exhibited a clear saturation tendency, where the growth rate of the boiling heat transfer coefficient decelerated sharply when the heat flux exceeded 250 kW·m^−2^. In contrast, the TiO_2_@MWCNT composite nanofluid sustained a high growth rate even in the high heat flux regime (q > 250 kW·m^−2^) without significant performance decay, which attests to its enhanced heat transfer stability during prolonged high-power operation—an effect attributed to the synergistic thermal conduction of MWCNTs and the hydrophilicity-induced bubble dynamic optimization of TiO_2_ nanoparticles.

At heat flux densities below 250 kW·m^−2^, the heat transfer coefficient (HTC) of the modified TiO_2_@MWCNT composite nanofluid increased sharply with rising heat flux density. Beyond this range, the HTC growth rate gradually diminished. A higher bubble nucleation density at low-to-medium heat flux densities enhanced liquid rewetting of the heating surface and suppressed adjacent bubble coalescence, avoiding continuous vapor film formation and sustaining efficient heat exchange. Meanwhile, intensified nanoparticle motion reduced the base fluid’s surface tension, lowering bubble growth resistance and promoting rapid bubble detachment from the heating surface—this maintained boiling regime stability. As the heat flux density approached the critical heat flux (CHF), heat transfer enhancement efficiency saturated, further slowing the HTC growth rate. Once the heat flux density exceeded 450 kW·m^−2^, the system entered the film boiling regime, where the heating surface was persistently wrapped by a continuous vapor film, severely hindering heat exchange. The HTC then showed no significant variation with increasing heat flux density, and boiling heat transfer became unstable.

## 4. Boiling Visualization Analysis

To investigate the boiling behavior of modified TiO_2_@MWCNT composite nanofluids, a high-speed imaging system was employed to capture the boiling process and quantify the nanofluid’s effects on bubble dynamics. The results demonstrate that the as-prepared composite nanofluid achieved optimal heat transfer performance at a modified TiO_2_: MWCNTs mass ratio of 2:1. However, owing to the poor optical transparency of the 2:1 nanofluid, a 3:1 mass ratio composite nanofluid (which exhibited analogous bubble dynamics) was chosen for high-speed imaging characterization.

**Figure 9:**
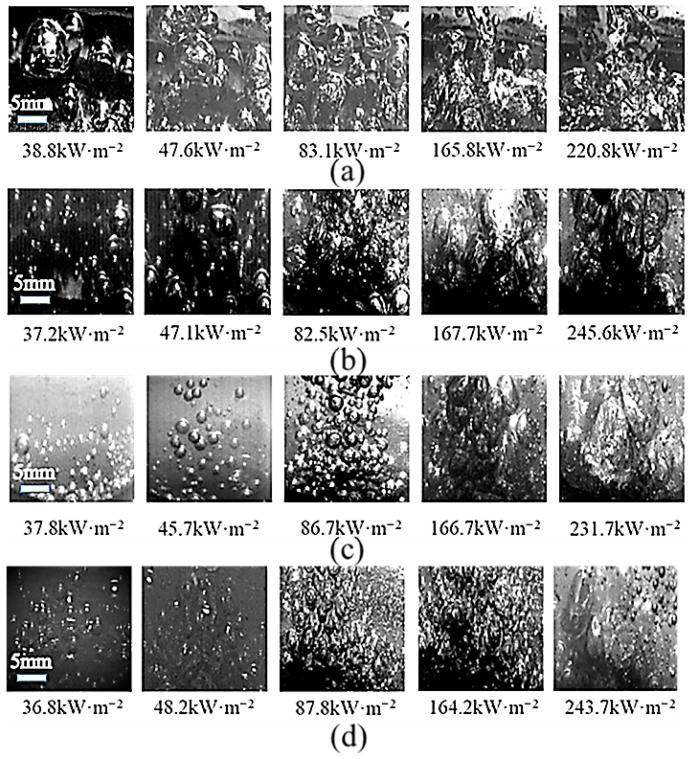
Composite nanofluid boiling images: (**a**) boiling state of deionized water; (**b**) boiling state of 0.05% wt TiO_2_ nanofluid; (**c**) boiling state of 0.05% wt modified TiO_2_ nanofluid; (**d**) boiling state of modified TiO_2_ nanofluid@multi-walled carbon nanotube (3:1) composite nanofluid.

Bubble dynamics in DI water, single-component modified TiO_2_ nanofluid, and composite nanofluid are compared in Figure 9. At the same heat flux density, the composite nanofluid exhibited a significantly higher bubble nucleation count than both DI water and the single-component nanofluid; its optimized bubble dynamics substantially enhanced boiling heat transfer performance. During the initial boiling stage, modified TiO_2_ nanoparticles enhanced the hydrophobicity of the heating surface, lowering the bubble nucleation barrier and markedly reducing the initial superheat. Simultaneously, numerous nucleation cavities on the heating surface were activated, thereby effectively increasing the bubble nucleation density and enhancing liquid rewetting of the heating surface. Theoretical analysis based on multiphase flow models [43], the Young–Laplace equation [44], and Carey’s correlation [45] (Equations (8)–(11)) demonstrates that an increased contact angle in composite nanofluids significantly enhances capillary suction (Pc), thereby accelerating the liquid rewetting process. Simultaneously, optimizing the contact angle can modulate the dynamic processes of bubble nucleation and growth. Nucleation site density (vaporization core density) closely correlates with the surface roughness of the heating surface, surface tension, and degree of superheat. The bubble detachment frequency is determined by the sum of bubble residence time and growth time and is the reciprocal of this combined time. This theoretically validates the intrinsic mechanism underlying the enhancement of boiling heat transfer.

(12) $$P_{c}=\frac{2\sigma cos\theta }{R}$$

(13) $$N_{a}=({r_{s}(\frac{h_{fg}\rho_{v}}{{2\sigma T}_{sat}})∆T_{sat})}^{m}$$

(14) $$D_{d}=0.0208\theta \sqrt{\frac{\sigma }{g(\rho_{L}-\rho_{v})}}$$

(15) $$f=\frac{1}{t_{g}+t_{w}}$$

In the equation, σ represents the surface tension of the working fluid; θ represents the contact angle; g represents the gravitational acceleration; $h_{fg}$ represents the latent heat of vaporization; $r_{s}$ represents the micropore radius of the heating surface; m represents the empirical constant associated with nucleation site distribution; f represents the bubble detachment frequency; $t_{w}$ represents the bubble residence time.

As the heat flux density rose, both the bubble diameter and detachment frequency increased markedly. The composite nanofluid architecture simultaneously inhibited excessive bubble growth—preventing large bubbles from impeding heat transfer—and facilitated liquid rewetting of the heating surface, which in turn further boosted the heat transfer coefficient (HTC). Furthermore, hydrophobic nanoparticles optimized the surface free energy of the heating surface, effectively alleviating nanoparticle agglomeration and preserving the dynamic stability of boiling bubbles.

With a further rise in heat flux density, the interparticle collision frequency in the composite nanofluid escalated significantly. This heightened collision frequency amplified capillary action in the liquid phase, facilitating liquid rewetting of the heating surface and thus increasing bubble detachment frequency. Meanwhile, the synergistic effect of enhanced interparticle collisions and the intrinsic architecture of the composite nanofluid efficiently suppressed bubble coalescence, driving a substantial improvement in the critical heat flux (CHF). By contrast, deionized (DI) water yielded larger bubbles that were prone to coalescence and agglomeration, which undermined the maintenance of stable nucleate boiling conditions. Its CHF enhancement was therefore limited relative to that of the composite nanofluids, resulting in inferior overall boiling heat transfer performance.

## 5. Conclusions

In this study, hydrophobically modified TiO_2_ nanofluids were prepared, with the incorporation of multi-walled carbon nanotubes (MWCNTs) to fabricate composite nanofluids featuring outstanding hydrophobicity and dispersibility. The resultant composite nanofluids were subjected to boiling heat transfer performance evaluation, while bubble dynamics were analyzed via a CCD high-speed camera system.

Experimental findings demonstrate that the incorporation of hydrophobically modified TiO_2_ nanoparticles and multi-walled carbon nanotubes (MWCNTs) significantly enhanced boiling heat transfer performance compared to single-component TiO_2_ nanofluids. At a mass ratio of hydrophobically modified TiO_2_ nanoparticles to MWCNTs of 2:1, the composite nanofluid achieved a 31.6% increase in heat transfer coefficient (HTC) and a 46.5% improvement in critical heat flux (CHF) relative to the single-component TiO_2_ nanofluid.

In composite nanofluids, multi-walled carbon nanotubes (MWCNTs) possess high thermal conductivity and an interconnected porous network structure. Hydrophobically modified TiO_2_ nanoparticles optimize the dispersion stability of the nanofluid and augment nucleation site density, constituting the core mechanism underlying heat transfer enhancement in composite nanofluids. During boiling, the formation of a nanoparticle-deposited thin film with a porous composite structure increases active nucleation sites, enhances capillary suction, promotes liquid rewetting toward the heating surface, and optimizes the overall boiling heat transfer process.

Bubble dynamics analysis demonstrates that uniformly dispersed hydrophobically modified TiO_2_@MWCNT composite nanofluids augment the density of active vaporization nuclei on heating surfaces, lower the energy barrier for bubble nucleation, facilitate bubble detachment and growth dynamics, and thereby enhance pool boiling heat transfer performance.

## Data Availability

Dataset available on request from the authors.
